# *S100a16* Deficiency Prevents Alcohol-induced Fatty Liver Injury via Inducing MANF Expression in Mice

**DOI:** 10.7150/ijbs.84472

**Published:** 2023-10-02

**Authors:** Dan Wang, Rihua Zhang, Xiaoxuan Qin, Jizheng Wang, Yifang Hu, Shan Lu, Jingbao Kan, Yaoqi Ge, Ke Jin, Wen-Song Zhang, Yun Liu

**Affiliations:** 1 Department of Geriatrics, The First Affiliated Hospital of Nanjing Medical University, Nanjing, Jiangsu, 210029, China.; 2 Department of Neurology, The First Affiliated Hospital of Nanjing Medical University, Nanjing, Jiangsu, 210029, China.; 3 Department of Infectious Diseases, The First Affiliated Hospital of Nanjing Medical University, Nanjing, Jiangsu, 210029, China.; 4 Department of Pharmacy, The First Affiliated Hospital of Nanjing Medical University, Nanjing, Jiangsu, 210029, China.; 5 Department of Medical Informatics, School of Biomedical Engineering and Informatics, Nanjing Medical University, Nanjing, Jiangsu, China.

**Keywords:** Alcoholic liver disease, S100A16, hepatic steatosis, MANF, ER stress.

## Abstract

Alcoholic liver disease (ALD) encompasses conditions ranging from simple steatosis to cirrhosis and even liver cancer. It has gained significant global attention in recent years. Despite this, effective pharmacological treatments for ALD remain elusive, and the core mechanisms underlying the disease are not yet fully comprehended. S100A16, a newly identified calcium-binding protein, is linked to lipid metabolism. Our research has discovered elevated levels of the S100A16 protein in both serum and liver tissue of ALD patients. A similar surge in hepatic S100A16 expression was noted in a Gao-binge alcohol feeding mouse model. *S100a16* knockdown alleviated ethanol-induced liver injury, steatosis and inflammation. Conversely, *S100a16* transgenic mice showed aggravating phenomenon. Mechanistically, we identify mesencephalic astrocyte-derived neurotrophic factor (MANF) as a regulated entity downstream of* S100a16* deletion. MANF inhibited ER-stress signal transduction induced by alcohol stimulation. Meanwhile, MANF silencing suppressed the inhibition effect of *S100a16* knockout on ethanol-induced lipid droplets accumulation in primary hepatocytes. Our data suggested that *S100a16* deletion protects mice against alcoholic liver lipid accumulation and inflammation dependent on upregulating MANF and inhibiting ER stress. This offers a potential therapeutic avenue for ALD treatment.

## Introduction

Alcohol-associated liver disease (ALD) has emerged as a significant global public health concern, exerting immense pressure on healthcare systems 1-3. Alcoholic fatty liver, the initial stage of ALD, is marked by hepatic steatosis, a process which has been identified as reversible. However, persistent alcohol intake can escalate this to more severe conditions such as alcoholic steatohepatitis, fibrosis, cirrhosis, and even hepatocellular carcinoma 4. Alarmingly, over 50% of cirrhosis-related mortalities globally are linked to alcohol abuse 3. To date, the precise mechanisms of ALD and effective interventions are yet to be fully elucidated 5. This highlights the pressing necessity to uncover the disease's underlying pathogenesis and pave the way for novel therapeutic strategies.

The S100 calcium-binding protein 16 (S100A16), a recently identified member of the EF-hand S100 family proteins, exhibits broad expression across diverse human tissues, notably in the liver and adipose tissue 6. In previous studies, we pinpointed S100A16 as a novel factor that promotes lipogenesis in the livers of high fat diet (HFD)-fed mice 7. Additionally, the knockdown of* S100a16* has shown potential in mitigating fatty liver injury. Our data further indicated that silencing of *S100a16* can protect mice from liver fibrosis when exposed to stimuli such as CCl4, bile duct ligation surgery, and methionine choline deficiency diet feeding 8. Moreover, a recent investigation revealed the protective effects of *S100a16* knockdown against lipid accumulation and inflammation in HK-2 cells under high glucose conditions 9. These findings underscore the potential role of S100A16 in the regulation of hepatic lipid metabolism and liver injury. Yet, its role in ALD's progression remains uncharted.

Endoplasmic reticulum stress (ER stress) is a cellular defense mechanism triggered in response to the accumulation of misfolded and unfolded proteins within the endoplasmic reticulum's lumen. This protective process initiates the unfolded protein responses (UPR). Three canonical UPR signaling pathways have been delineated: inositol-requiring protein-1 (IRE1), activating transcription factor-6 (ATF6), and protein kinase RNA (PKR)-like ER kinase (PERK) 10, 11. Notably, the pathological link between ER stress and ALD has been substantiated through both experimental and clinical studies 12-15.

Mesencephalic astrocyte-derived neurotrophic factor (MANF) is an emerging dopaminergic neurotrophic factor believed to have regulating potential for ER stress 16, 17. It has been confirmed that MANF is also a key regulator in hepatic lipid metabolism 18. Hepatocyte-specific *Manf* overexpression could directly promote browning of white adipocytes via the p38 MAPK pathway, thereby counteracting HFD-induced obesity 19. In addition, mice hepatocyte-specific *Manf* overexpression reduced lipid accumulation in liver, whereas *Manf* ablation exacerbated hepatic steatosis. Mechanistic insights revealed that MANF inhibition upregulated G0S2, a critical determinant that fostered lipid accumulation and suppressed lipolysis 19, 20.

In our current research, we observed a marked upregulation of S100A16 in ALD patients. Additionally,* S100a16* knockdown mitigated the severity of alcohol-induced fatty liver injury in mice. Mechanistically, the surge in S100A16 induced by alcohol was found to inhibit MANF, which in turn fostered lipid accumulation and inflammatory reactions. Collectively, our findings posited S100A16 as a potential novel regulator in the pathogenesis of ALD.

## Material and Methods

### Human samples

Pathological section of liver and serum from subjects with ALD and healthy controls were obtained from the first affiliated hospital of Nanjing Medical University (Nanjing, China). All ALD patients had history of alcohol consumption averaging at least 70 g/day, for at least 20 years. We excluded patients with drug-induced liver injury, autoimmune liver disease, hepatitis B or C, and hepatocellular carcinoma. Samples from relative healthy subjects were known to have no history of excessive drinking or chronic liver disease. The information of ALD patients and healthy individuals are shown in Table 1. All research was conducted in accordance with both the Declarations of Helsinki and Istanbul. The study protocol and use of bio-samples for research were approved by the ethics committee of the First Affiliated Hospital of Nanjing Medical University (No.2022-SR-582).

### Animal experiments

*S100a16* knockout heterozygote (*S100a16*^KO+/-^) mice and *S100a16* transgenic (*S100a16*^TG^) mice were generated as described previously 7, 8. Briefly, the *S100a16* allele with floxed exon 2-3 was backcrossed into the C57BL/6 background. It was then bred with EIIa-Cre mice expressing the Cre recombinase to generate *S100a16*^KO+/-^ in over 98% C57BL/6 background. During this process, we found that* S100a16* homozygous (*S100a16*^KO-/-^) mutations were lethal. So, we used *S100a16* knockout heterozygote (*S100a16*^KO+/-^) mice in this research.

*S100a16* mice cDNA was cloned into the pCAGGS plasmid, transgenic mice (*S100a16*^TG^ mice) were generated by purified DNA fragments microinjection into the pronuclei of fertilized eggs of C57BL/6J.

Hepatocyte-specific *S100a16* knockout (*S100a16*^LKO^) mice were generated by crossing *S100a16*-floxed mice with Albumin-Cre transgenic mice (Alb-Cre) with both strains on the C57BL/6J background. *S100a16*-floxed (*S100a16*^f/f^) mice were mated with Alb-Cre mice to generate *S100a16*^LKO^ heterozygote mice. Then, *S100a16*^LKO^ mice and their littermate controls (*S100a16*^f/f^ mice) were obtained by mating *S100a16*^LKO^ heterozygote mice with *S100a16*^f/f^ mice.

All mice used in our experiments were identified by PCR analyses of the genomic DNA obtained from tail tips. All animal were housed in ventilated cages at the SPF facility in a temperature-controlled environment with a 12-hour light/dark cycle and had free access to water and diet. All animal experiments were approved by the Institutional Animal Care and Use Committee of Nanjing Medical University (No. IACUC-2105059).

### Ethanol feeding studies

Age-matched eight- to ten-week-old male mice were treated with either an isocaloric ethanol or pair-fed control diet following the protocol of NIAAA model 21. Briefly, all mice were first fed ad libitum with the control Lieber-DeCarli liquid diet (Bio-Serv, product No. F1259SP) for 5 days, Ethanol groups were then fed with a liquid diet (Bio-Serv, product No. F1258SP) containing 5% (vol/vol) ethanol for 10 days and control groups were fed with isocaloric control diet for 10 days. At day 11, mice were administrated by oral gavage in the morning with a single bolus of ethanol (5 g/kg body weight) or isocaloric maltose dextrin solution (9 g/kg body weight), respectively. The mice were sacrificed and blood and tissue samples were collected 9 h post-gavage. Liquid diets were freshly prepared from powder daily. Food intake was recorded daily. Depilation, refusal to eat, intolerable pain, and dramatic weight loss were used as humane endpoints in this experiment.

Serum and liver triglyceride (TG), total cholesterol (T-CHO) and serum alanine transaminase (ALT), aspartate transaminase (AST) levels were determined using biochemical assay kits (Nanjing Jiancheng Bioengineering Institute, A110-1-1, A111-1-1, C009-2-1 and C010-2-1) according to the manufacturer's specifications.

### Cell culture and treatment

Mice primary hepatocytes were isolated by *in situ* perfusion digestion of livers using type IV collagenase (Gibco, 17104019), followed by separation using PERCOLL (sigma, P1644) and centrifugation. Isolated primary hepatocytes were maintained in DMEM medium supplemented with 10 % Fetal Bovine Serum (FBS, Gibco, 10270-106) and 1 % penicillin/streptomycin (Gibco, 15140122). The culture medium was replaced daily.

AML12 hepatocytes, a non-transformed mouse hepatocyte cell line, was maintained in DMEM/F12 medium (Gibco, 11330-032) supplemented with ITS liquid media supplement (Sigma, I3146), 40 ng/mL dexamethasone (Sigma, D4902-100mg) and 10 % FBS (Gibco, 10270-106). Cells were treated with medium containing 50 mM ethanol for 24 h and changed intervals fresh medium at 12 h. All the cells were incubated at 37 °C in a humidified atmosphere containing 5% CO_2_.

### Gene silencing and overexpression

For the knockdown and overexpression of *S100a16* and *Manf*, the AML12 cell line was transfected with siRNA or a full-length plasmid using Lipofectamine® 2000 transfection reagent (Invitrogen, 11668019) according to the manufacturer's protocol. Knockdown and overexpression of *Manf* in *S100a16*^LKO^ or *S100a16*^TG^ mice primary hepatocytes were performed as above described. Negative scrambled control siRNA and empty vector plasmid (pcDNA3.1) were transfected into the control groups.

### Protein isolation and Western blotting

Total protein was extracted from liver tissues and cells using RIPA lysis buffer (Beyotime P0013) added with protease inhibitors (biosharp BL612A) and phosphatase inhibitors (biosharp BL615A). Protein samples were mixed with loading buffer (Beyotime P0298) and separated by 6-12.5% sodium dodecyl sulfate-polyacrylamide gel electrophoresis. Then, the proteins were transferred onto nitrocellulose membranes (BioTrace^TM^ NT T33517). Nitrocellulose membranes were blocked in TBST containing 5% skim milk for 2 h at room temperature. Then the membranes were incubated in primary antibodies overnight at 4 ℃, followed by incubation in the corresponding secondary antibodies. The relative expression levels of target proteins were quantified using ImageJ software (version 1.48). All primary antibodies used are listed in Table S1.

### RNA isolation and RT-qPCR analysis

Total mRNA was extracted using RNA easy Isolation Reagent (Vazyme, R701-01). The concentration of RNA was quantified by a Nanodrop2000 spectrophotometer (Thermo Scientific, USA). cDNA was synthesized using HiScript III RT SuperMix for qPCR (Vazyme, R323-01) and detected using QuantStudio 7 system (Applied Biosystems). Relative mRNA expression levels were calculated using 2^-ΔΔct^ method and were normalized against the levels of 18s. The primer sequences used for each gene are listed in Table S2.

### Histological Examination (HE) staining

Fresh liver tissue was fixed in 4 % formaldehyde solution. Fixed liver tissues were embedded in paraffin, cut into 5 μm thick slices and stained with hematoxylin and eosin (H&E) following the standard protocol for analysis.

### Oil Red O staining

Fresh liver tissue was embedded with OCT freeze embedding agent and frozen, and then cut into 5 μm thick slices. Oil-red was stained following the standard protocol for analysis. Slices were scanned by an automatic digital slide scanner.

### BODIPY staining

Primary hepatocytes and AML 12 cells were first fixed with 4% paraformaldehyde followed by incubation with BODIPY® 493/503 (Invitrogen 2295013) for lipid staining. Cell nuclei were counterstained with DAPI (SouthernBiotech, H0621-V341). The lipid droplets were visualized under a THUNDER Imaging Systems (Leica, THUNDER DMi8).

### Immunofluorescence

Liver tissue slices were permeabilized with 0.5 % Triton X-100 for 30 min (after deparaffinization) and blocked with 5 % goat serum for 1 h at RT. Then the slices were incubated with anti-S100A16 or anti-MANF primary antibodies overnight at 4°C, followed by an appropriate fluorescently tagged secondary antibody in the dark at RT for 1.5 h. Primary and secondary antibodies were diluted in 3 % BSA. DAPI was used to stain nuclei. Fluorescence microscopy was performed using a THUNDER Imaging Systems (Leica, THUNDER DMi8).

### ELISA

Commercially available human ELISA kits (CUSABIO, Wuhan, China) were used to detect S100A16 levels in human serum (ALD and healthy individuals) according to the instructions provided by the manufacturer.

### RNA sequencing and analysis

Primary hepatocytes (n=3/group) were isolated from alcohol-fed WT and *S100a16*^KO+/-^ mice. Total RNA was extracted, and mRNA was purified. mRNA was fragmented and reverse transcribed to generate cDNA. Sequencing analysis was performed as our previous work 8. Genes with *p* value < 0.05 and fold change ≥ 2 were considered differentially expressed using DESeq2 software between *S100a16* silencing group and WT group. Gene functional annotations were assessed based on Kyoto Encyclopedia of Genes and Genomes (KEGG) and Gene Ontology (GO) databases. The raw sequence data have been submitted to the NCBI Gene Expression Omnibus (GEO) database (accession no. GSE243683).

### Statistical analysis

All data are expressed as the mean ± SEM. Two-tailed student's t-test was used to calculate the statistical significance between two groups and one-way analysis of variance (one way ANOVA) for three or more groups. In all cases, *p* < 0.05 was considered statistically significant.

## Results

### Alcohol consumption up-regulated S100A16 expression in human and mice

**Figure 1:**
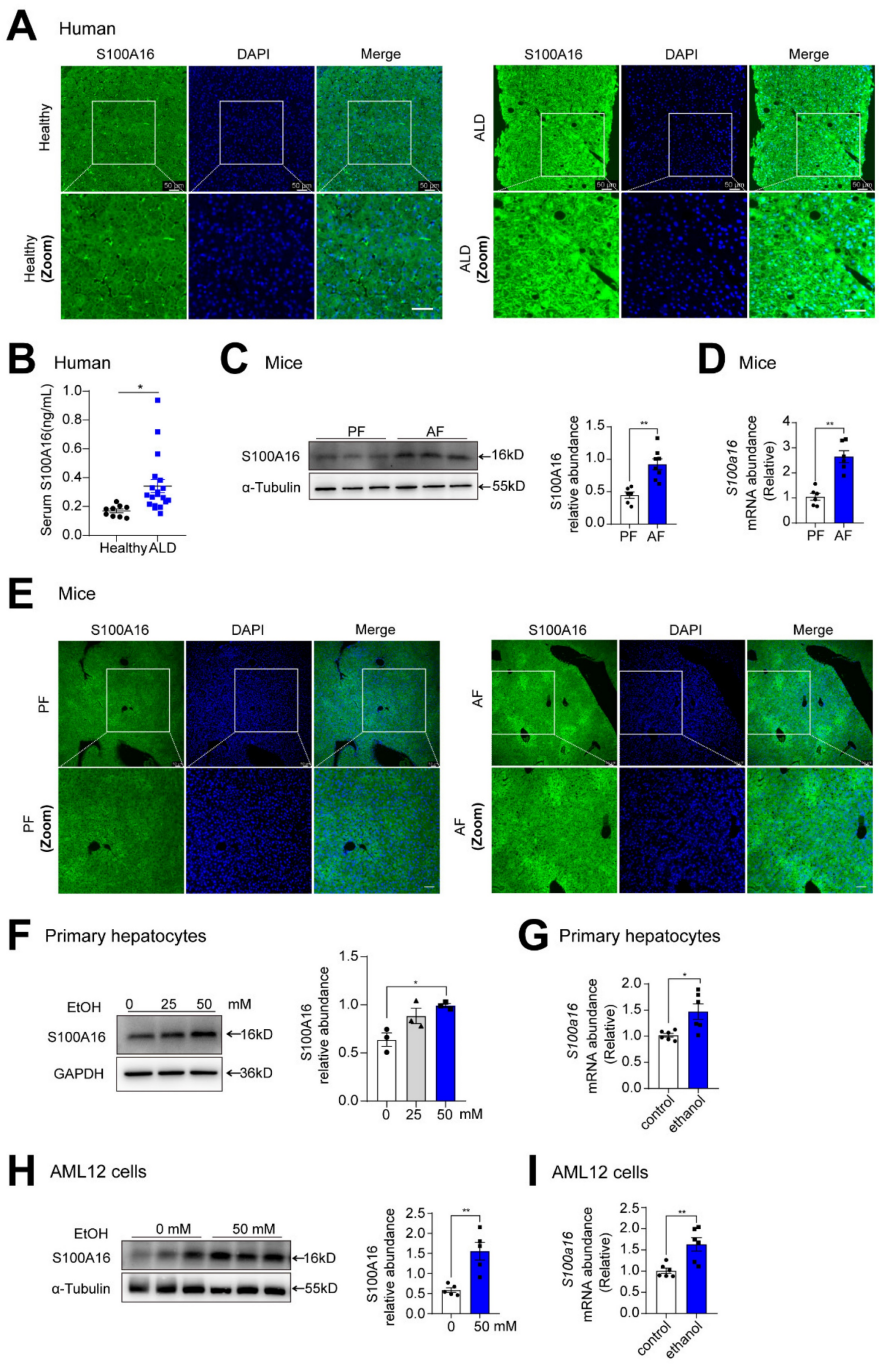
** Alcohol consumption up-regulated S100A16 expression in human and mice.** (A) Representative immunofluorescence staining of S100A16 in liver paraffin slices from healthy controls or patients with alcoholic-associated liver disease (ALD). Scale bar = 50 μm. (B) ELISA analysis of S100A16 expression in serum of ALD patients(n=19) and healthy controls(n=9). (C-E) Mice were pair-fed or alcohol-fed for 10 d and administered a single binge of ethanol (NIAAA model). (C) Western blot analysis of S100A16 expression in mice liver. Quantification of western blots on the right. (D) *S100a16* mRNA levels in mice liver. (E) Immunofluorescence assessment of S100A16 expression in mice liver. (F-G) Protein and mRNA expression of S100A16 in primary hepatocytes treated with ethanol (50 mM) for 24 h. (H-I) Protein and mRNA expression of S100A16 in AML 12 cells treated with ethanol (50 mM) for 24 h. α-Tubulin or GAPDH served as the loading control. All data are represented as the mean ± SEM values. *: *p* < 0.05 *vs*. the control group, **: *p* < 0.01 *vs*. the control group. ALD: alcohol-associated liver disease. PF: pair-fed group. AF: alcohol-fed group.

To investigate the potential involvement of S100A16 in ALD progression, we first evaluated S100A16 expression levels in patients with ALD and healthy controls. Both immunofluorescence and immunohistochemical assays indicated S100A16 was significantly increased in the ALD liver tissue compared with healthy individuals **(Fig. 1A, S1A)**. Concurrently, serum S100A16 levels were found to be elevated in individuals diagnosed with ALD **(Fig. 1B)**. Subsequently, we employed a short-term chronic plus one binge ethanol feeding model (NIAAA model). Analyses using western blot, RT-qPCR, and immunofluorescence staining collectively highlighted the heightened expression of S100A16 in the livers of alcohol-fed WT mice **(Fig. 1C-E)**. In parallel, *in vitro* experiments illustrated that ethanol exposure upregulated S100A16 expression in primary hepatocytes as well as AML12 cells **(Fig. 1F-I)**. Overall, these results suggested that alcohol consumption significantly increased hepatic S100A16 expression.

### *S100a16* deletion protected mice against alcohol-induced fatty liver injury

**Figure 2:**
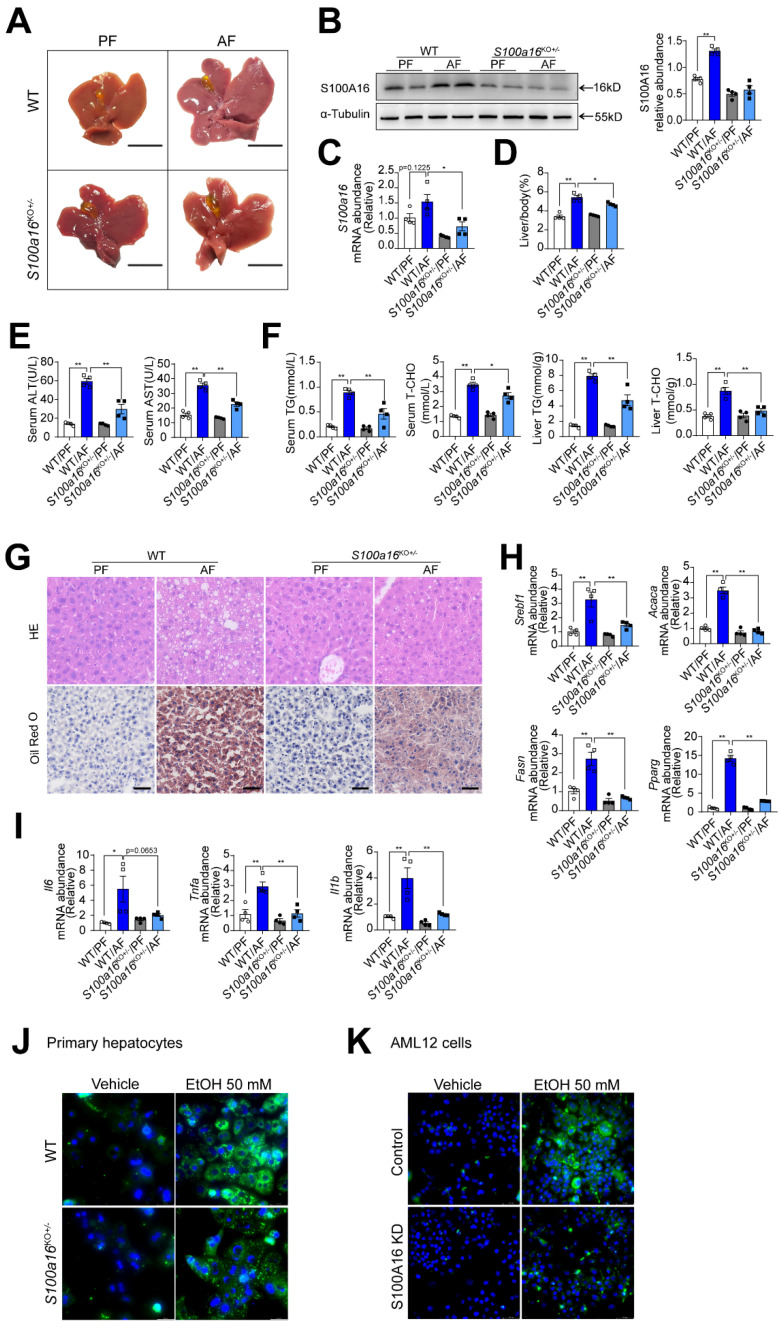
***S100a16* deletion protected mice against alcohol-induced fatty liver injury.** WT or *S100a16*^KO+/-^ mice were fed separately with the corresponding liquid diet 10 days and administered a single binge of ethanol. (A) Pictures of mice liver from different groups. Protein (B) and mRNA (C) levels of S100A16 in the liver from WT and *S100a16*^KO+/-^ mice. Quantification of western blots on the right. (D) Liver weight/body weight (%). (E) Serum ALT, AST levels. (F) Serum TG, TC levels and liver TG, TC levels. (G) Representative H&E and Oil Red O staining of mice livers. Scale bars: 50 µm. (H) Relative mRNA levels of *Srebf1*, *Acaca*, *Fasn*, and *Pparg*. (I) Relative mRNA levels of *Il6*, *Tnfa*, and *Il1b*. (J) Primary hepatocytes from WT and *S100a16^KO^*^+/-^mice were treated with ethanol (50 mM) for 24 h. Representative images of BODIPY 493/503 staining of lipid droplets. Scale bars: 50 µm. (K) AML 12 cells with knockdown of S100A16 by siRNA (S100A16 KD) were treated with ethanol (50 mM) for 24 h. Representative images of BODIPY 493/503 staining of lipid droplets. Scale bars: 50 µm. α-Tubulin served as the loading control. All data are represented as the mean ± SEM values. *: *p* < 0.05 *vs*. the control group, **: *p* < 0.01 *vs*. the control group. ALT, alanine aminotransferase; AST, aspartate aminotransferase; TG: triacylglycerol; T-CHO: total cholesterol; H&E, hematoxylin and eosin.

To elucidate the relationship between S100A16 and alcoholic fatty liver disease, we generated *S100a16* knockout mice. Homozygous deletion of *S100a16* in mice causes mortality, therefore we used *S100a16* heterozygous knockout mice (*S100a16*^KO+/-^ mice). Genotyping of *S100a16*^KO+/-^ and wild type (WT) mice were performed before the experiment **(Fig. S2A)**. Deletion of S100A16 in the liver of *S100a16*^KO+/-^ mice was confirmed at the protein level **(Fig. S2B)**. Our data showed that the livers became bigger in the WT group after ethanol exposure, but this condition was alleviated in alcohol-fed *S100a16*^KO*+/-*^ mice **(Fig. 2A)**. Western blot and RT-qPCR assays verified the augmented S100A16 expression in the livers of alcohol-fed WT mice **(Fig. 2B-C)**. While body weights remained consistent between alcohol-fed *S100a16*^KO+/-^ and WT mice **(Fig. S2C)**, but liver/body weight ratios were mitigated in *S100a16*^KO*+/-*^ group **(Fig. 2D)**. As shown in **Fig. 2E**, knockout of *S100a16* alleviated the upregulation of serum ALT, AST levels during alcohol consumption. Serum TG, TC contents and liver TG, TC contents all showed a decline in the *S100a16*^KO+/-^ group **(Fig. 2F)**. Liver histological studies, encompassing H&E and Oil Red O staining, pointed to a mitigated liver injury in the *S100a16*^KO+/-^ cohort **(Fig. 2G)**. Additionally, there was a marked suppression in the expression of crucial lipogenic and inflammatory genes in the livers of these mice upon genetic *S100a16* inhibition **(Fig. 2H-I)**. Also noteworthy, lipid droplets in ethanol-induced primary hepatocytes isolated from *S100a16*^KO+/-^ mice showed a significant reduction when compared with that from WT mice **(Fig. 2J)**. As shown in **Fig. S2D**, we employed siRNA to diminish S100A16 expression (S100A16 KD) in AML12 cells and the knockdown efficiency was confirmed using western blot. Analogous to our earlier findings, *S100a16* knockdown led to diminished lipid droplets in AML12 cells **(Fig. 2K)**. In summary, inhibiting S100A16 substantially alleviated the symptoms of alcohol-induced fatty liver injury.

### *S100a16* overexpression aggravated alcohol-induced fatty liver injury in mice

**Figure 3:**
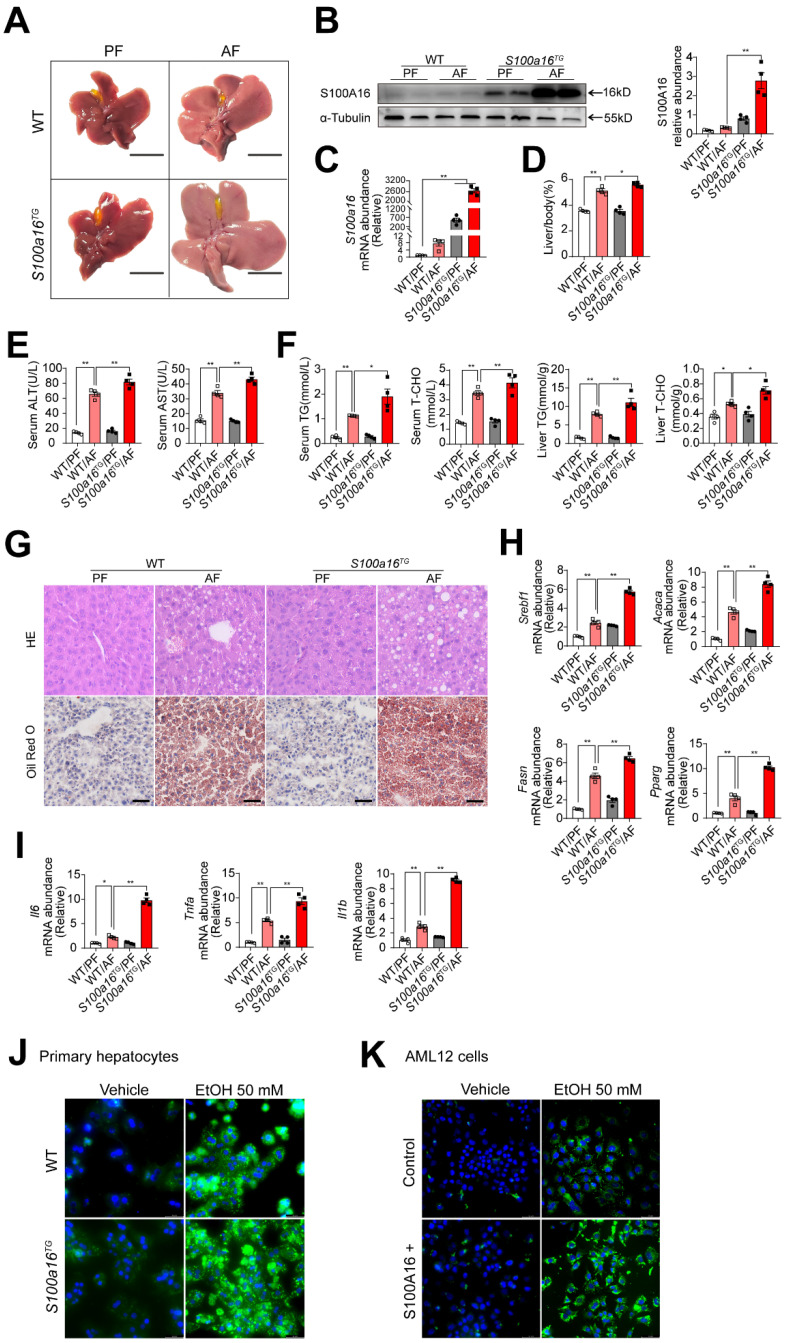
***S100a16* overexpression aggravated alcohol-induced fatty liver injury in mice.** WT or *S100a16*^TG^ mice were fed separately the corresponding liquid diet 10 days and administered a single binge of ethanol. (A) Pictures of mice liver from different groups. Protein (B) and mRNA (C) levels of S100A16 in the liver from WT and *S100a16*^TG^ mice. Quantification of western blots on the right. (D) Liver weight/body weight (%). (E) Serum ALT, AST levels. (F) Serum TG, TC levels and liver TG, TC levels. (G) Representative H&E and Oil Red O staining of mice livers. Scale bars: 50 µm. (H) Relative mRNA levels of *Srebf1*, *Acaca*, *Fasn*, and *Pparg*. (I) Relative mRNA levels of *Il6*, *Tnfa*, and *Il1b*. (J) Primary hepatocytes from WT and *S100a16*^TG^ mice were treated with ethanol (50 mM) for 24 h. Representative images of BODIPY 493/503 staining of lipid droplets. Scale bars: 50 µm. (K) AML 12 cells with overexpression of S100A16 by overexpression plasmid (S100A16+) were treated with ethanol (50 mM) for 24 h. Representative images of BODIPY 493/503 staining of lipid droplets. Scale bars: 50 µm. α-Tubulin served as the loading control. All data are represented as the mean ± SEM values. *: *p* < 0.05 *vs*. the control group, **: *p* < 0.01 *vs*. the control group.

To further verify the relationship between S100A16 and alcoholic fatty liver injury diseases, *S100a16* transgenic (*S100a16*^TG^) mice systemically overexpressing *S100a16* were engineered under control of the pCAGGS vector promoter **(Fig. S3A)**. Expression of S100A16 in the liver of *S100a16*^TG^ mice was confirmed at the protein level **(Fig. S3B)**. We then exposed age-matched WT and *S100a16*^TG^ mice to the NIAAA model, observing the subsequent changes. Unlike the results from *S100a16*^KO+/-^ mice, post-ethanol exposure, the livers of *S100a16*^TG^ mice had larger livers than WT mice after ethanol exposure **(Fig. 3A)**. Moreover, liver-to-body weight ratios surged in the *S100a16*^TG^ group **(Fig. 3D)**. Results showed that S100A16 overexpression in liver was increased in the alcohol-fed mice **(Fig. 3B-C)**. Contrasting with the effects of *S100a16* suppression, overexpression of *S100a16* exacerbated the progression of alcohol-induced fatty liver development compared with WT mice. This exacerbation was evident both biochemically (assessing ALT, AST, TG, TC levels) and histologically (using H&E and Oil Red O staining) **(Fig. 3E-G)**. The mRNA levels of molecules that promote fat accumulation and inflammation were also augmented in alcohol-exposed *S100a16*^TG^ mice **(Fig. 3H-I)**. Notably, ethanol-induced primary hepatocytes derived from *S100a16*^TG^ mice exhibited a pronounced increase in lipid droplets compared with primary hepatocytes from WT mice **(Fig. 3J)**. As shown in **Fig. S3C**, S100A16 overexpression efficiency in AML 12 cells was confirmed by western blot analysis. Similarly, overexpression of S100A16 in AML12 cells also amplified ethanol-induced lipid accumulation **(Fig. 3K)**. Collectively, these findings compellingly argued that overexpressing S100A16 intensified the manifestations of alcohol-induced hepatic steatosis and injury.

### *S100a16* deficiency upregulated MANF expression in alcohol-fed mice

**Figure 4:**
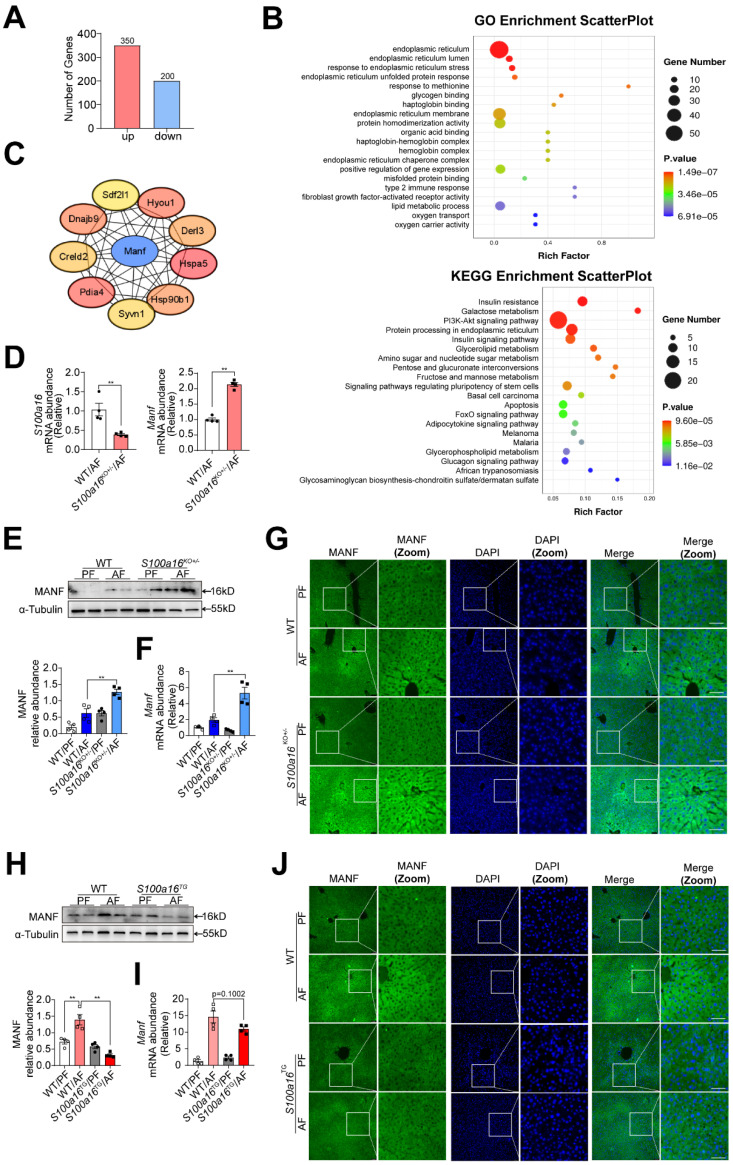
***S100a16* deficiency upregulated MANF expression in alcohol-fed mice.** (A-C) Primary hepatocytes(n=3/group) were isolated from alcohol-fed WT and *S100a16*^KO+/-^ mice and genome-wide analysis of gene expression was performed by transcriptome sequencing. (A) The number of up-regulation (350) and down-regulation(200) of differentially expressed genes (p ≤ 0.05, fold change ≥ 2). (B) GO and KEGG pathways enrichment analyses. (C) Hub genes of differentially expressed genes. We constructed a protein-protein interaction network and identified hub genes related to alcoholic fatty liver using STRING and Cytoscape. (D) mRNA levels of *S100a16* and *Manf* in primary hepatocytes from WT and *S100a16*^KO+/-^ mice after alcohol feeding. (E) protein and(F) mRNA levels of MANF in the alcohol-fed WT and *S100a16*^KO+/-^ mice livers. Quantification of western blots on the right. (G)Representative immunofluorescence images of MANF in the alcohol-fed WT and *S100a16*^KO+/-^ mice livers. Scale bar: 50 μm. (H) protein and(I) mRNA levels of MANF in the alcohol -fed WT or *S100a16*^TG^ mice livers. Quantification of western blots on the right. (J) Representative immunofluorescence images of MANF in the alcohol -fed WT and *S100a16*^TG^ mice livers. Scale bar:50 μm. α-Tubulin served as the loading control. All data are represented as the mean ± SEM values. *: *p* < 0.05 *vs*. the control group, **: *p* < 0.01 *vs*. the control group. MANF: mesencephalic astrocyte-derived neurotrophic factor.

Subsequently, we examined the underlying mechanism of S100A16 in regulating alcoholic fatty liver injury. Primary hepatocytes were isolated from alcohol-fed WT or *S100a16*^KO+/-^ mice subjected to NIAAA model and genome-wide analysis of gene expression was performed by transcriptome sequencing. Those detectable genes with a fold change ≥ 2 and p value < 0.05 were defined as differentially expressed genes, when compared with equivalents measured from WT cells. Consequently, *S100a16*^KO+/-^ cells gave rise to 550 differentially expressed genes (350 upregulated and 200 downregulated, **Fig. 4A**). The PCA plot is presented in **Fig. S4A**. Utilizing GO and KEGG enrichment analyses, we discerned the distribution of these differentially expressed genes, uncovering that the absence of *S100a16* significantly affected intracellular processes, including the endoplasmic reticulum stress (ER stress) pathway **(Fig. 4B)**. We then constructed a protein-protein interaction network **(Fig. S4B)** and identified hub genes using STRING (https://stringdb.org/) and Cytoscape (version 3.8.0). Notably, among these hub genes **(Fig. 4C)**, mesencephalic astrocyte-derived neurotrophic factor (MANF) has been implicated as pivotal in alcohol-induced liver diseases 22, 23. Emerging studies have portrayed MANF as an ER-resident protein influencing various metabolic diseases, including alcoholic fatty liver 18, 19. As shown in **Fig. 4D,** we verified the RNA sequencing result using RT-qPCR. In mice liver tissue, we also found that gene and protein expression of MANF were increased in WT mice after alcohol feeding and these alterations were further increased in *S100a16*^KO+/-^ mice** (Fig. 4E-G)**. Conversely, a decline in MANF gene and protein expression was observed in alcohol-fed *S100a16*^TG^ mice **(Fig. 4H-J)**. From these findings, we postulated that *S100a16* suppression ameliorated alcoholic fatty liver injury by augmenting MANF expression.

To more accurately confirm the role of S100A16 in alcoholic fatty liver disease, *S100a16*^f/f^ mice were crossed with Albumin-Cre mice to generate hepatocyte-specific S100A16 knockout mice (genotype: *S100a16*^LKO^) and control mice (genotype: *S100a16*^f/f^) **(Fig. S5A-B)**. Deletion of S100A16 in the liver of *S100a16*^LKO^ mice was confirmed at the protein level **(Fig. S5C)**. mRNA and protein levels of MANF were increased in the livers from alcohol-fed* S100a16*^LKO^ mice compare with* S100a16*^f/f^ mice **(Fig. S5D-E)**. Similar with *S100a16*^KO+/-^ mice, liver/body weight ratios were alleviated in *S100a16*^LKO^ group **(Fig. S5F)**. As depicted in **Fig. S5G-I**, the specific knockout of S100A16 in hepatocytes attenuated alcohol-induced fatty liver development, as demonstrated by both biochemical assay (ALT, AST, TG, TC levels) and histological examination (H&E staining). We also observed that hepatocyte-specific genetic suppression of *S100a16* significantly curtailed the expression of key lipogenic and inflammatory genes in mice livers **(Fig. S5J-K).**

### S100A16 restrained MANF and activated ER stress in ALD

**Figure 5:**
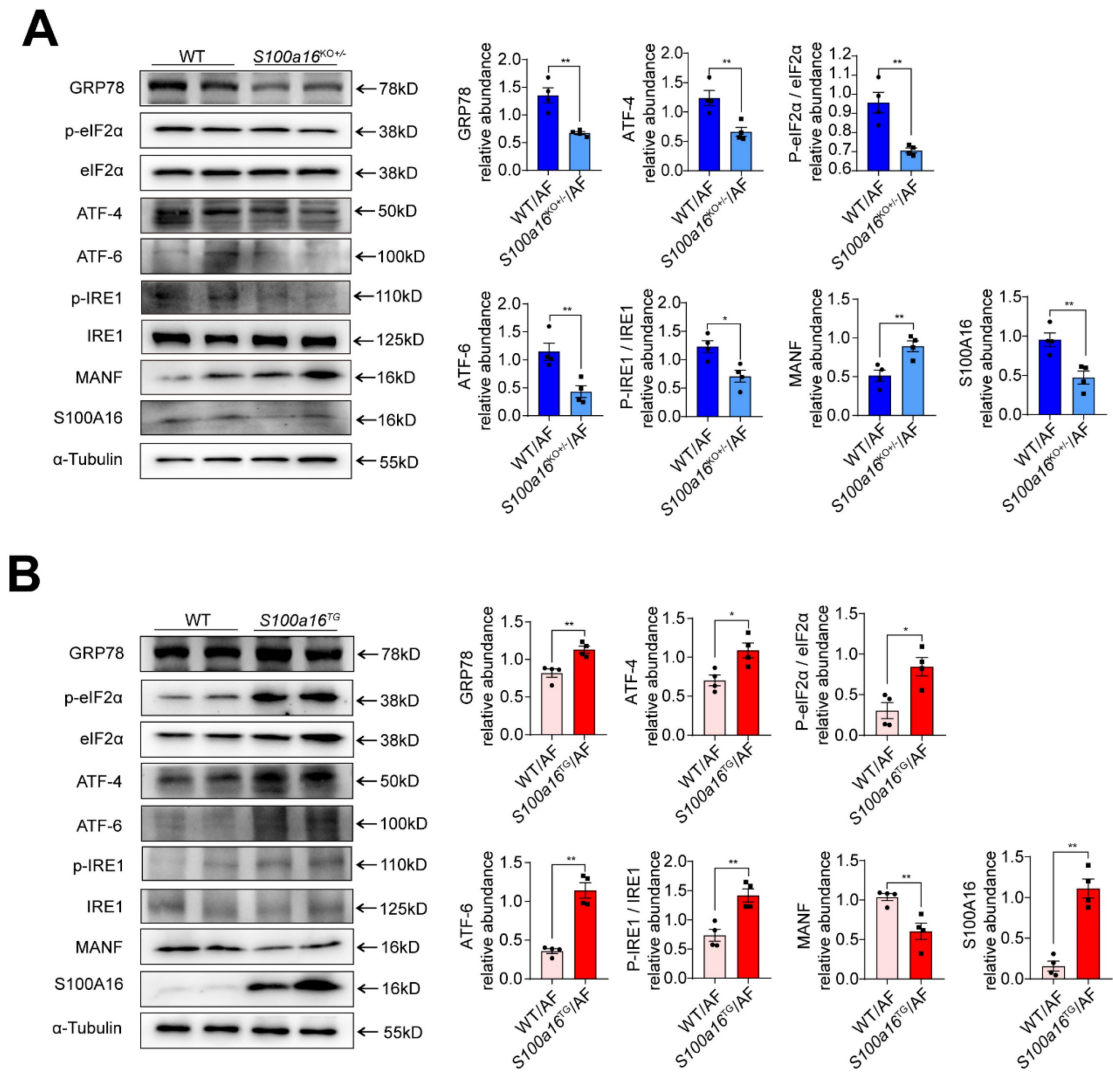
** S100A16 restrained MANF and activated ER stress in ALD.** (A) Western blot analysis of the S100A16, MANF and the ER stress-related genes GRP78, p-IRE1α, IRE1α, ATF6, ATF4, p-EIF2α, EIF2α in the livers of alcohol-fed WT or *S100a16*^KO+/-^ mice. Quantification of western blots on the right. (B) Western blot analysis of the S100A16, MANF and the ER stress-related genes in the livers of alcohol-fed WT or *S100a16*^TG^ mice. Quantification of western blots on the right. α-Tubulin served as the loading control. All data are represented as the mean ± SEM values. *: *p* < 0.05 *vs*. the control group, **: *p* < 0.01 *vs*. the control group. ATF4: activating transcription factor-4; ATF6: activating transcription factor-6; EIF2: eukaryotic initiation factor 2; GRP78: glucose-regulated protein 78; IRE1: inositol-requiring protein-1.

MANF, situated within the ER lumen, serves as a crucial shield against ER stress-induced cellular damage 24. Given previous reports that ER stress is intricately linked with the pathogenesis of alcohol-induced fatty liver disease 15, we examined the expression of typical factors related to ER stress. Our findings revealed that, in alcohol-exposed *S100a16*^KO+/-^mice livers, there was a pronounced reduction in the expression of ER stress markers such as GRP78, ATF4, ATF6, and in the phosphorylation levels of IRE1α and EIF2α compared with their WT counterparts **(Fig. 5A)**. Conversely, these trends were inverted in the *S100a16*^TG^ mice **(Fig. 5B)**. In summation, *S100a16* ablation augmented MANF expression, thereby curtailing ER stress, which in turn mitigates the progression of alcoholic fatty liver development.

### S100A16 regulated alcohol-induced lipid accumulation dependent on altering MANF expression

**Figure 6:**
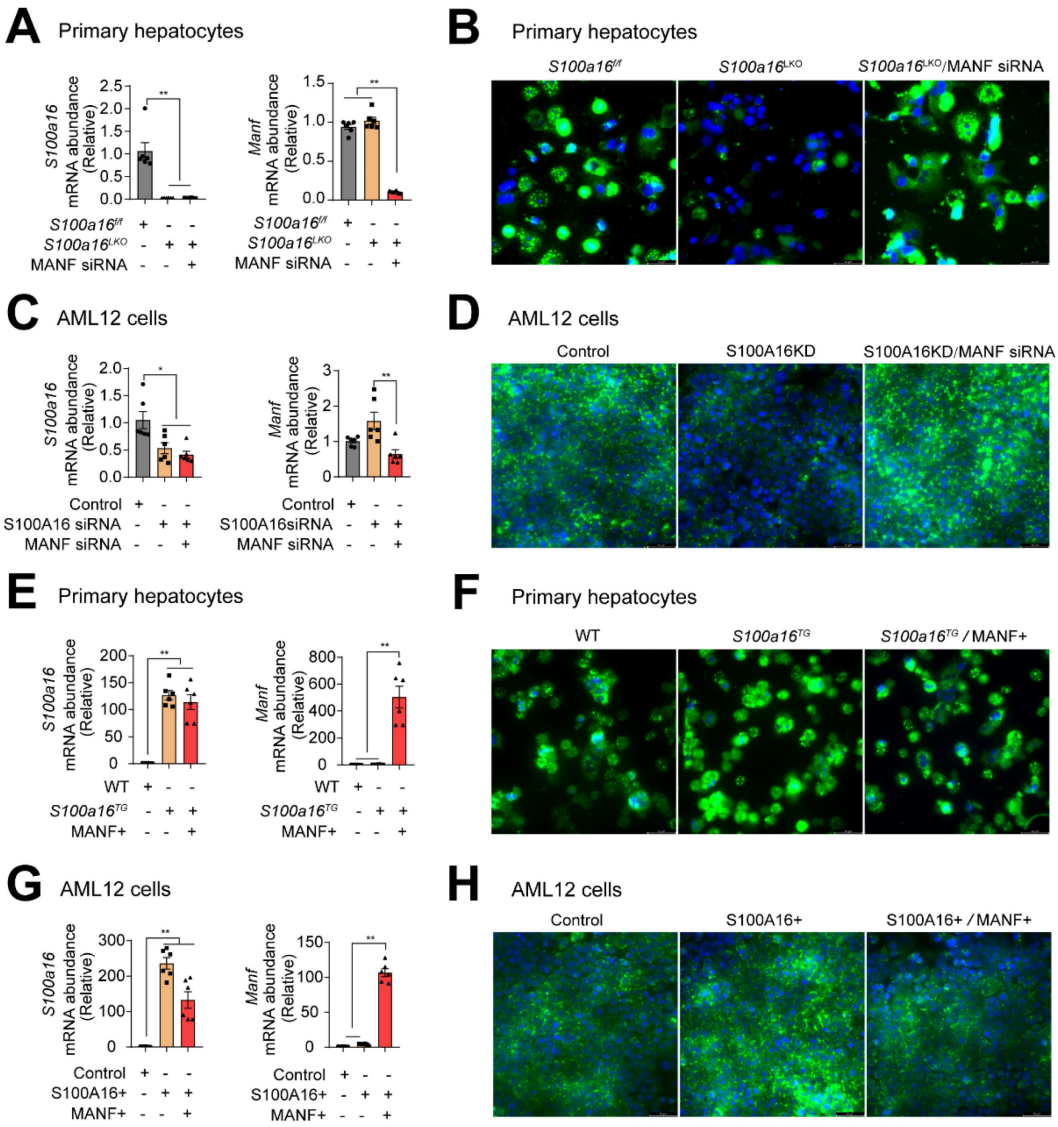
** S100A16 regulated alcohol-induced lipid accumulation dependent on altering MANF expression.** (A) mRNA levels of *S100a16* and *Manf* in ethanol-induced primary hepatocytes from *S100a16*^f/f^ or *S100a16*^LKO^ mice transfected with *Manf* siRNA. (B) Representative BODIPY staining images are shown on the right side, Scale bar: 50 μm. (C) mRNA levels of *S100a16* and *Manf* in ethanol-induced AML 12 cells co-transfected with *S100a16* and *Manf* siRNA. (D) Representative BODIPY staining images are shown on the right side, Scale bar: 50 μm. (E) mRNA levels of *S100a16* and *Manf* in ethanol-induced primary hepatocytes from WT or *S100a16*^TG^ mice transfected with overexpression plasmid. (F) Representative BODIPY staining images are shown on the right side, Scale bar: 50 μm. (G) mRNA levels of *S100a16* and *Manf* in ethanol-induced AML 12 cells co-transfected with S100A16 and MANF overexpression plasmid. (H) Representative BODIPY staining images are shown on the right side, Scale bar: 50 μm. All data are represented as the mean ± SEM values. *: *p* < 0.05 *vs*. the control group, **: *p* < 0.01 *vs*. the control group. *S100a16*^LKO^: hepatocyte-specific *S100a16* knockout.

To ascertain whether S100A16 mediated ethanol-induced lipid accumulation dependent on MANF, we silenced MANF in primary hepatocytes isolated from *S100a16*^LKO^ mice. RT-qPCR analyses authenticated the deletion of both S100A16 and MANF in primary hepatocytes **(Fig. 6A)**. Observations from BODIPY staining indicated that* Manf* siRNA suppressed the inhibiting effects of *S100a16* deletion on fat accumulation **(Fig. 6B)**. Double knockdown of *Manf* and *S100a16* in AML12 cells also confirmed these results **(Fig. 6C-D)**. Elevated TG levels further substantiated that double knockdown of *Manf* and *S100a16* in primary hepatocytes/AML12 cells inhibited the lipid-reducing impact of *S100a16* deletion** (Fig. S6A-B)**. Conversely, we induced overexpression of *Manf* in primary hepatocytes from *S100a16*^TG^ mice, RT-qPCR analyses confirmed elevated levels of both *S100a16* and *Manf* **(Fig. 6E)**. Results showed that MANF overexpression significantly counteracted the lipid-enhancing effects of *S100a16* overexpression **(Fig. 6F)**. This observation was also corroborated in AML 12 cells **(Fig. 6G-H)**. TG levels showed the same results **(Fig. S6C-D)**. In essence, these outcomes suggested that the protective role of *S100a16* deletion protected hepatocytes against alcohol-induced lipid accumulation dependent on promoting MANF expression.

## Discussion

**Figure 7:**
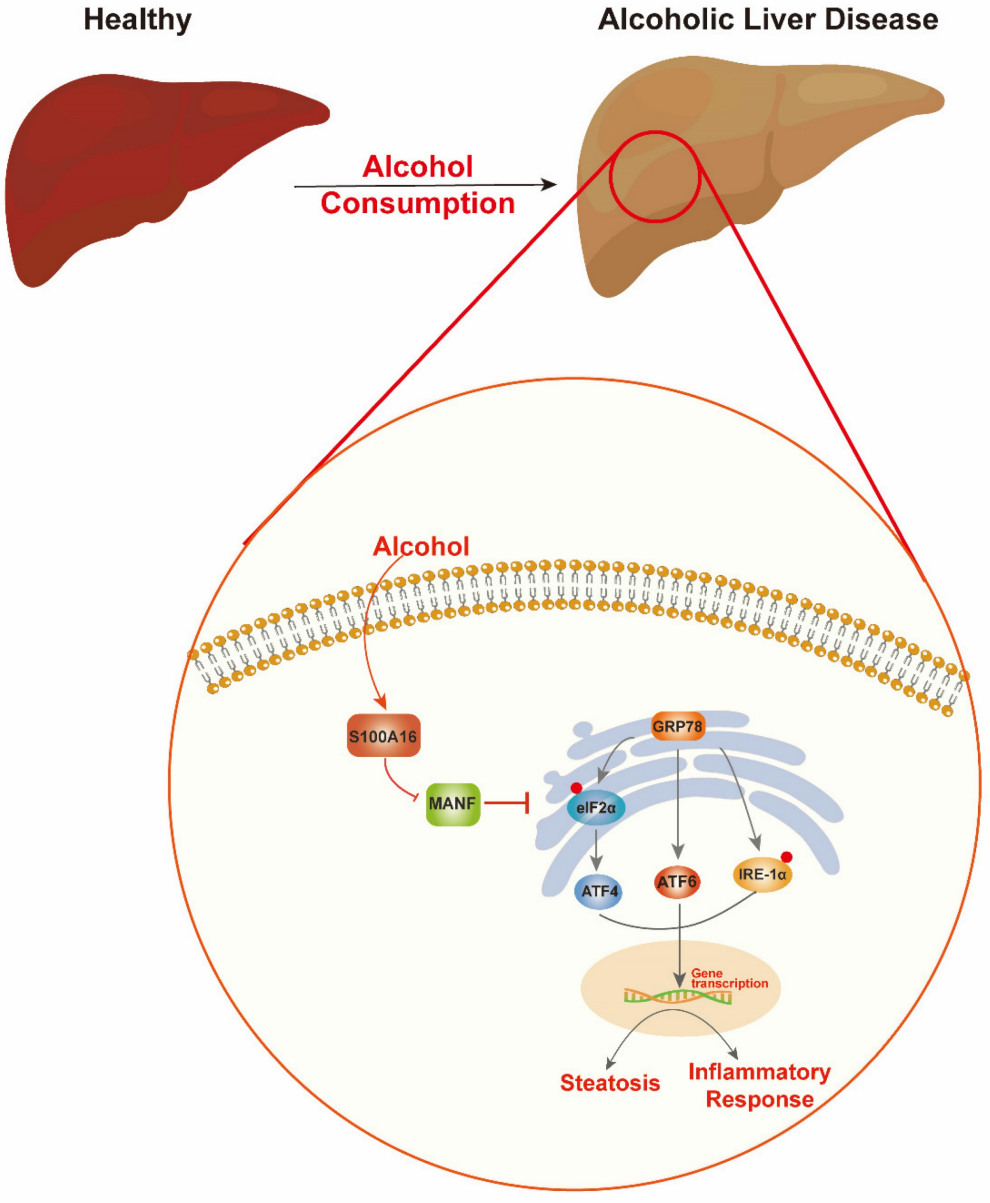
** Schematic representation of the role of S100A16 in the pathogenesis of ALD.** Alcohol consumption induced S100A16 upregulation. S100A16 aggravated ethanol-induced liver injury, steatosis and inflammation through inhibiting MANF expression and promoting ER stress-related signaling pathways.

Alcohol abuse stands as a prime cause of liver-related mortalities globally. Despite its grave implications, there remains an absence of efficacious treatments beyond lifestyle adjustments 5. This underscores the pressing need to identify innovative therapeutic targets for ALD. Our earlier investigations revealed that S100A16 modulated fatty liver development under high-fat diet conditions and high glucose-induced lipid accumulation 7, 9. Both ALD and NAFLD are progressive conditions transitioning from steatosis to steatohepatitis, fibrosis, and eventually cirrhosis. While they share certain facets, distinct clinical characteristics and molecular mechanics demarcate them. For instance, ALD is more characterized by inflammatory cell infiltration, whereas lipid droplet accumulation is predominant in NAFLD. Despite these differences, existing interventions for both ailments are limited 25, 26. Guided by our prior research, this study aimed to discern S100A16's role in ALD. Our findings revealed an up-regulation of S100A16 in both ALD patients and alcohol-exposed mice. S100A16 emerged as a catalyst for lipogenesis and inflammation, modulating MANF expression and ER stress signaling cascades** (Fig. 7)**.

S100 proteins traditionally influence myriad cellular functions, from differentiation and cycle progression to motility 27. Recent findings have also unveiled their role in liver lipid metabolism. Specifically, S100A8 aggravated hepatitis in high fat and cholesterol-fed mice 28, while S100A11 spurs hepatic steatosis via the RAGE-mediated AKT-mTOR signaling axis 29. Like most members of the S100 family, S100A16 was firstly reported as a regulatory factor related to malignant transformation and tumor development 6, 30-32. Despite these known effects of S100A16, its role in other diseases is just emerging. Our previous studies showed the association of S100A16 with adipogenesis 33, 34, NFALD 7 and liver fibrosis 8. Our current study underscored its upregulation in the liver and serum of ALD patients, suggesting its role in both ALD and NAFLD despite their different pathogenic mechanisms. These findings disclose an unrecognized mechanism for the role of S100A16 as a potential target for ALD and expose new insights into the action of other novel therapeutic targets for NAFLD to alleviate ALD progression.

Another major finding of this study is that we identified MANF, an endoplasmic reticulum-resident protein, as the downstream of S100A16 in alcoholic liver lipid accumulation. MANF is an emerging therapeutic target for metabolic diseases, including diabetes 35, obesity 19, fatty liver 20, alcohol-related diseases, and cardiovascular diseases 36. Hepatic MANF overexpression improved adipose inflammation, insulin sensitivity and hepatic steatosis in HFD-fed mice 19. Conversely, Hepatic MANF knockdown increased free fatty acid uptake and free fatty acid synthesis, which caused hepatic lipid accumulation and accelerated fatty liver development 20. MANF deficiency also accelerated lipogenesis and exacerbated lipid accumulation in HepG2 cells 37. In addition, MANF alleviated alcohol-induced hepatic steatosis via alleviating ER stress or activating STAT3-mediated autophagy 22, 23. These events indicated that MANF is highly expressed in liver and plays an important role in the regulation of liver disease. In this study, transcriptomics results showed a significant upregulation of MANF in primary hepatocytes isolated from *S100a16*^KO+/-^ mice compared with cells from WT. We further confirmed that MANF expression in liver was markedly promoted in alcohol-fed *S100a16*^KO+/-^ mice. Reverse results were observed in alcohol-fed *S100a16*^TG^ mice livers. Therefore, we speculated that S100A16 downregulated MANF expression and promoted alcohol-induced liver lipid accumulation and inflammation responses. In addition, MANF silencing abrogated the inhibitory effects of *S100a16* knockout on lipid droplets formation in primary hepatocytes isolated from *S100a16*^LKO^ mice in ethanol-containing medium. In contrast, MANF overexpression abrogated the protective effects of *S100a16* transgene on lipid droplet formation. Similar results were confirmed in AML12 cells cultured medium containing ethanol. These results provided evidences that *S100a16* deletion alleviates alcoholic fatty liver injury dependent on upregulating MANF expression. Besides, we also evaluated how S100A16 regulate MANF expression. We found S100A16 inhibited MANF expression using RNA sequencing, so we speculated that S100A16 transcriptionally regulated MANF. Indeed, S100A16 co-transfection decreased the luciferase activity of MANF promoter **(Fig. S6E)**. A cycloheximide (CHX) chase assay was performed in S100A16 plasmid-transfected AML 12 cells to investigate the effect of S100A16 on the degradation rate of MANF protein. When protein synthesis was inhibited with CHX to exclude the potential effects, MANF protein was degraded in a time-dependent manner and S100A16 overexpression resulted in faster degradation, indicating that S100A16 promoted MANF degradation **(Fig. S6F)**. Based on these results, we assumed that S100A16 inhibited MANF expression both by inhibiting its mRNA transcription and accelerating protein degradation process.

ER stress participated in the regulatory process of many diseases, including metabolic disorders 38, cancer 39 and neurological disorders 40. It has been reported that S100 family proteins play a specific role in regulating ER stress. S100A1 expression is inhibited by CTCF and activates the PERK signaling pathway of ER stress in cardiomyocytes 41. Hepatic steatosis and liver injury exacerbation of genetic mutations- or HFD-induced obesity is associated with an impaired UPR and persistent ER stress in the liver 42. In our previous work about renal tubulointerstitial fibrosis, we found that the interaction between S100A16 and GRP78 is mediated through the IRE1α pathway and activated ER stress 43. According to recent reports, ER stress upregulates NNMT through PERK-ATF4 pathway and promotes the development of ALD, indicating the role of ER stress in ALD pathogenesis 15. Consistent with these reports, we also found that S100A16 influenced MANF expression and regulated ER stress to aggravate alcohol-induced lipid metabolism. In conclusion, our data provided evidence that S100A16 is a novel regulatory factor in ALD. S100A16 transcriptionally downregulated MANF and aggravated ER stress to mediate ALD responses. These findings suggested the translational potential of S100A16 as a therapeutic target for ALD.

It has been reported that males are more likely than females to consume alcohol and males are diagnosed with alcohol use disorders more often than females 44. In addition, male and female have different reaction to alcohol consumption because of genetic patterns, hormone expression, and alcohol metabolism 45. Further studies are needed in order to understand the differences between male and female in ALD.

In summation, our insights underscore S100A16 as a novel ALD regulatory factor, affecting MANF expression and exacerbating ER stress. This points to S100A16's potential as a promising therapeutic bullseye for ALD.

## Supplementary Material

Supplementary materials and methods, figures and tables.

## Figures and Tables

**Table 1:** Demographic and clinical parameters of healthy controls and patients with ALD.

Variables	Healthy controls (n=9)	ALD patients (n=19)
Sex, male, n (%)	88.9	89.5
Age, y	52.6±3.9	58.3±2.2
ALT level, U/L	19.2±0.8	170.7±49.9*
AST level, U/L	21.5±0.7	150.7±29.2**
ALP level, U/L	80.2±6.5	174±26.8*
GGT level, U/L	23.5±2.1	232±50.5**
TBIL, μmol/L	11.3±0.82	191.1±44.4**
Albumin level, g/dL	45.4±0.6	31.4±1.2
hepatic malignancies	Negative	Negative
Alcohol Consumption, g/d	Negative	≥70

All the data are shown as mean ± SEM. * p < 0.05 vs. Control group. ** p < 0.01 vs. Control group.

## Abbreviations

AF: alcohol-fed group
ALD: alcohol-associated liver disease
ALT: alanine aminotransferase
AST: aspartate aminotransferase
ATF4: activating transcription factor-4
ATF6: activating transcription factor-6
EIF2: eukaryotic initiation factor 2
ER stress: endoplasmic reticulum stress
FBS: fetal bovine serum
GAPDH: glyceraldehyde-3-phosphatedehydrogenase
GRP78: glucose-regulated protein 78
HE: hematoxylin and eosin
IRE1: inositol-requiring protein-1
MANF: mesencephalic astrocyte-derived neurotrophic factor
PF: pair-fed group
RT-qPCR: quantitative real-time polymerase chain reaction
S100A16: S100 calcium binding protein 16
*S100a16* ^LKO^: hepatocyte-specific *S100a16* knockout
TG: triglyceride
T-CHO: total cholesterol
UPR: unfolded protein responses
WT: wild type
