# Supplementary material for: S100a16 Deficiency Prevents Alcohol-induced Fatty Liver Injury via Inducing MANF Expression in Mice
Source: Int J Biol Sci. 2023 Oct 2;19(16):5074–88. doi: 10.7150/ijbs.84472 (PMC10620815; doi:10.7150/ijbs.84472)
Supplement: Supplementary file 1 — Supplementary materials and methods, figures and tables. [file ijbsv19p5074s1.pdf]

Supplementary materials

S100a16 Deficiency Prevents Alcohol-induced Fatty Liver Injury via Inducing MANF Expression in Mice

Dan Wang[a#], Rihua Zhang[a#], Xiaoxuan Qin[b], Jizheng Wang[a], Yifang Hu[a], Shan Lu[a], Jingbao Kan[a], Yaoqi Ge[a], Ke Jin[c*], Wen-Song Zhang[a,d*], Yun Liu[a,e*]

Affiliations:
[a] Department of Geriatrics, The First Affiliated Hospital of Nanjing Medical University, Nanjing, Jiangsu, 210029, China
[b] Department of Neurology, The First Affiliated Hospital of Nanjing Medical University, Nanjing, Jiangsu, 210029, China
[c] Department of Infectious Diseases, The First Affiliated Hospital of Nanjing Medical University, Nanjing, Jiangsu, 210029, China
[d] Department of Pharmacy, The First Affiliated Hospital of Nanjing Medical University, Nanjing, Jiangsu, 210029, China
[e] Department of Medical Informatics, School of Biomedical Engineering and Informatics, Nanjing Medical University, Nanjing, Jiangsu, China.

#: These authors contributed equally to this work.
*: Correspondence author:
Yun Liu, Department of Geriatrics, The First Affiliated Hospital of Nanjing Medical University, 300 Guangzhou Road, Nanjing, 210029, China. Email address: liuyun@njmu.edu.cn.
Wen-Song Zhang, Department of Pharmacy, The First Affiliated Hospital of Nanjing Medical University, 300 Guangzhou Road, Nanjing, 210029, China. Email address: zhangwensong91@126.com.
Ke Jin, Department of Infectious Diseases, The First Affiliated Hospital of Nanjing Medical University, 300 Guangzhou Road, Nanjing, 210029, China. Email address: penghaoren2001@126.com.

Keywords:

Alcoholic liver disease; S100A16; hepatic steatosis; MANF; ER stress.

Material and Methods

Immunohistochemistry (IHC)

Mouse liver samples were fixed, embedded in paraffin, and sectioned at 5 μm thickness for IHC. IHC staining was performed with anti-S100A16 antibody. The primary antibody was incubated at 4 °C overnight, and washed three times with PBS for 10 min. Finally, a horseradish peroxidase-conjugated secondary antibody is applied.

Mouse tail genotyping

Commercially available Mouse Direct PCR Kit (Bimake, B40013) were used to detect mouse tail genotyping according to the instructions provided by the manufacturer， and then agarose gel electrophoresis.

Luciferase reporter gene analysis

Control (pcDNA 3.1) or S100A16 overexpression plasmid was co-transfected with MANF promoter luciferase reporter gene in AML 12 cells. After 48 h, the cells were lysed, and luciferase activities were measured with the Dual-Luciferase Reporter Assay System (Promega, E1910) using an automatic microplate reader (BioTek,Synergy H1). Renilla luciferase plasmid was used as an internal control.

Statistical analysis

All of the experimental results were statistically analyzed using GraphPad Prism 8.0 software. All data are expressed as the mean ± SEM. Two-tailed student's t-test was used to calculate the statistical significance between two groups and one-way analysis of variance (one way ANOVA) for three or more groups. In all cases, $p < 0.05$ was considered statistically significant.

Supplementary Figures

A

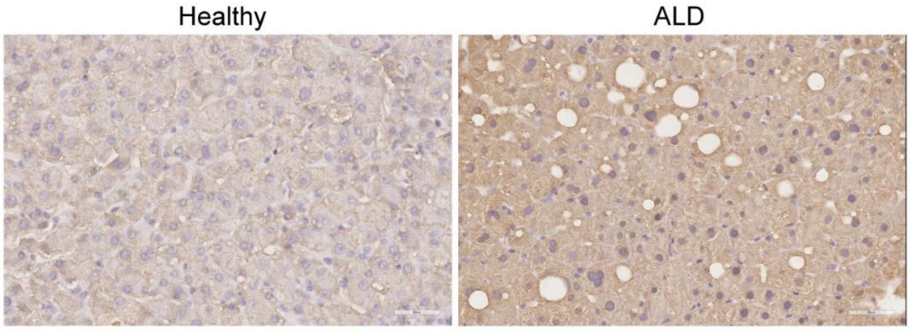

Fig.S1 (A) Representative immunohistochemical staining images of S100A16 in liver paraffin slices from healthy controls and patients with alcoholic-associated liver disease (ALD). Scale bar :50 μm.

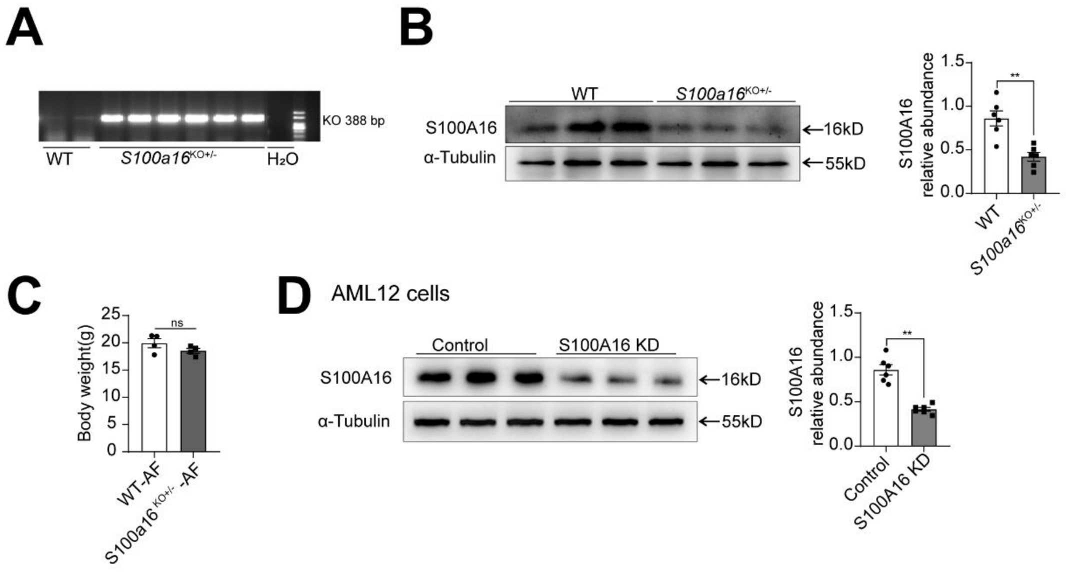

Fig.S2 (A) A representative gel electrophoresis image for genotyping. (B) Western blot analyses of S100A16 in the liver from WT and *S100a16*$^{KO+/-}$ mice. (C) Body weight from WT and *S100a16*$^{KO+/-}$ mice after alcohol feeding. (D) Western blot analyses of S100A16 in AML 12 cells after transfection by siRNA (S100A16 KD). α-Tubulin served as the loading control. All data are represented as the mean ± SEM values. *: $p < 0.05$ *vs*. the control group, **: $p < 0.01$ *vs*. the control group.

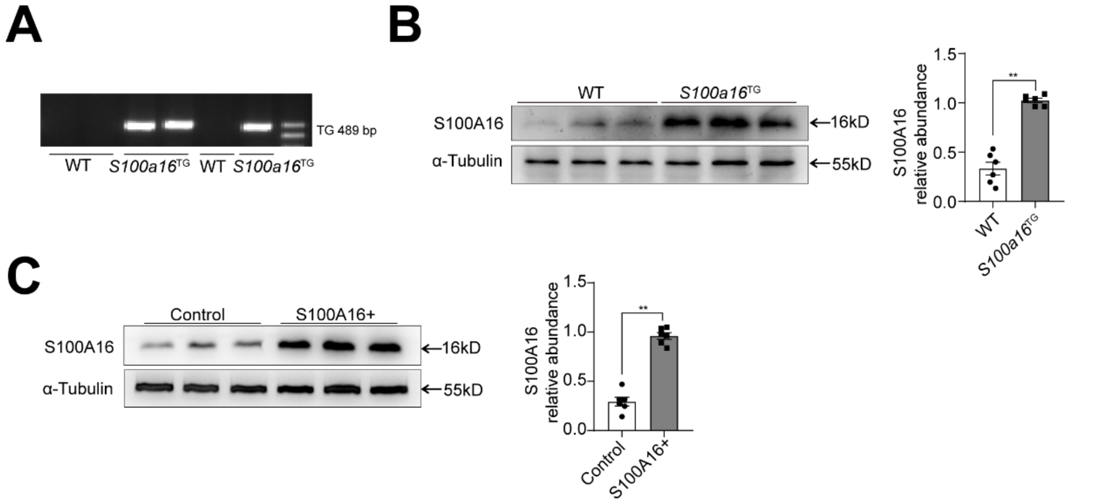

Fig.S3 (A) A representative gel electrophoresis image for genotyping. (B) Western blot analyses of S100A16 in the liver from WT and *S100a16*^{TG} mice. (C) Western blot analyses of S100A16 in AML 12 cells after transfection by overexpression plasmid (S100A16+). α-Tubulin served as the loading control. All data are represented as the mean ± SEM values. *: $p < 0.05$ *vs*. the control group, **: $p < 0.01$ *vs*. the control group.

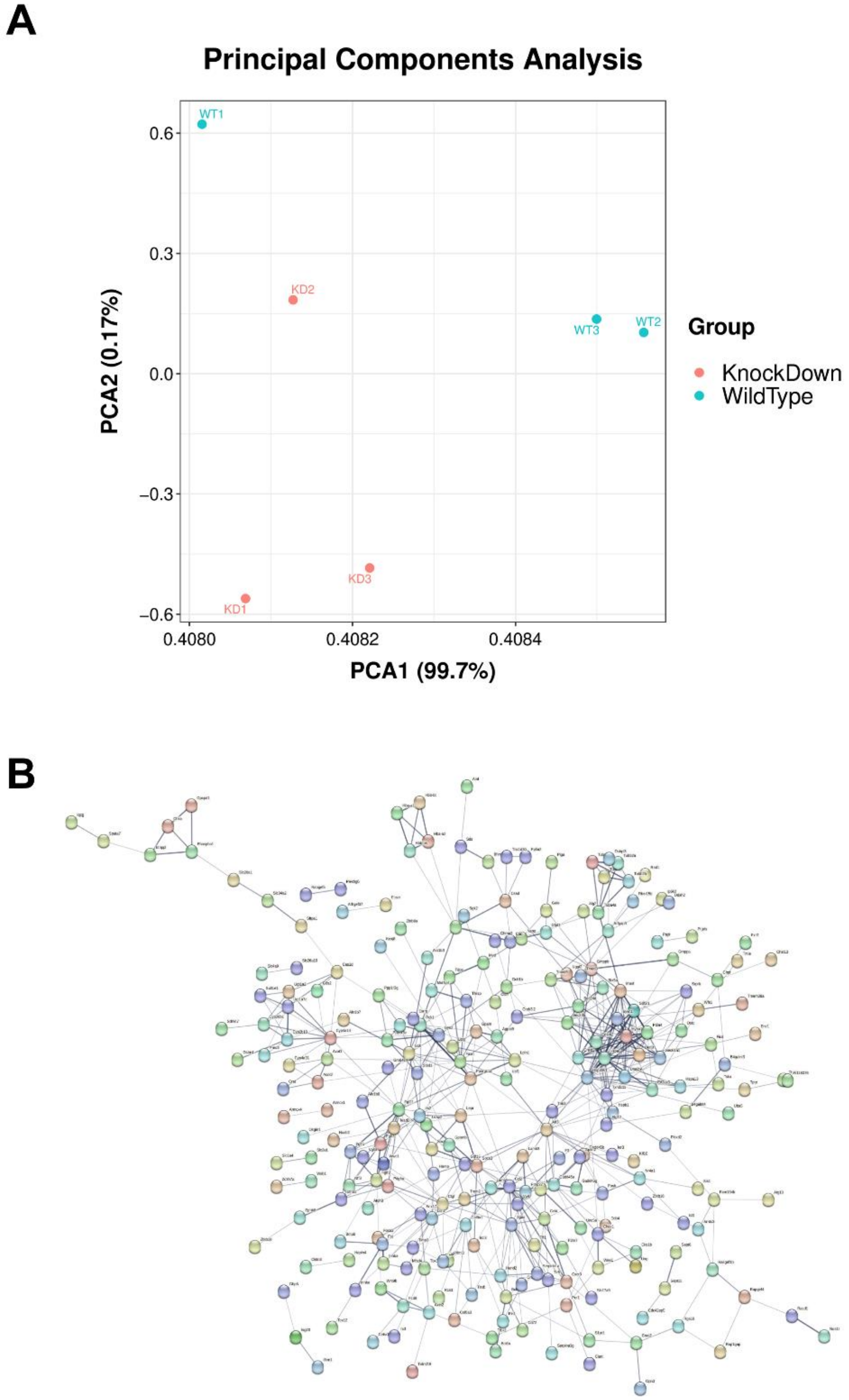

Fig.S4 (A) A PCA plot. (B) Protein-protein interaction network picture.

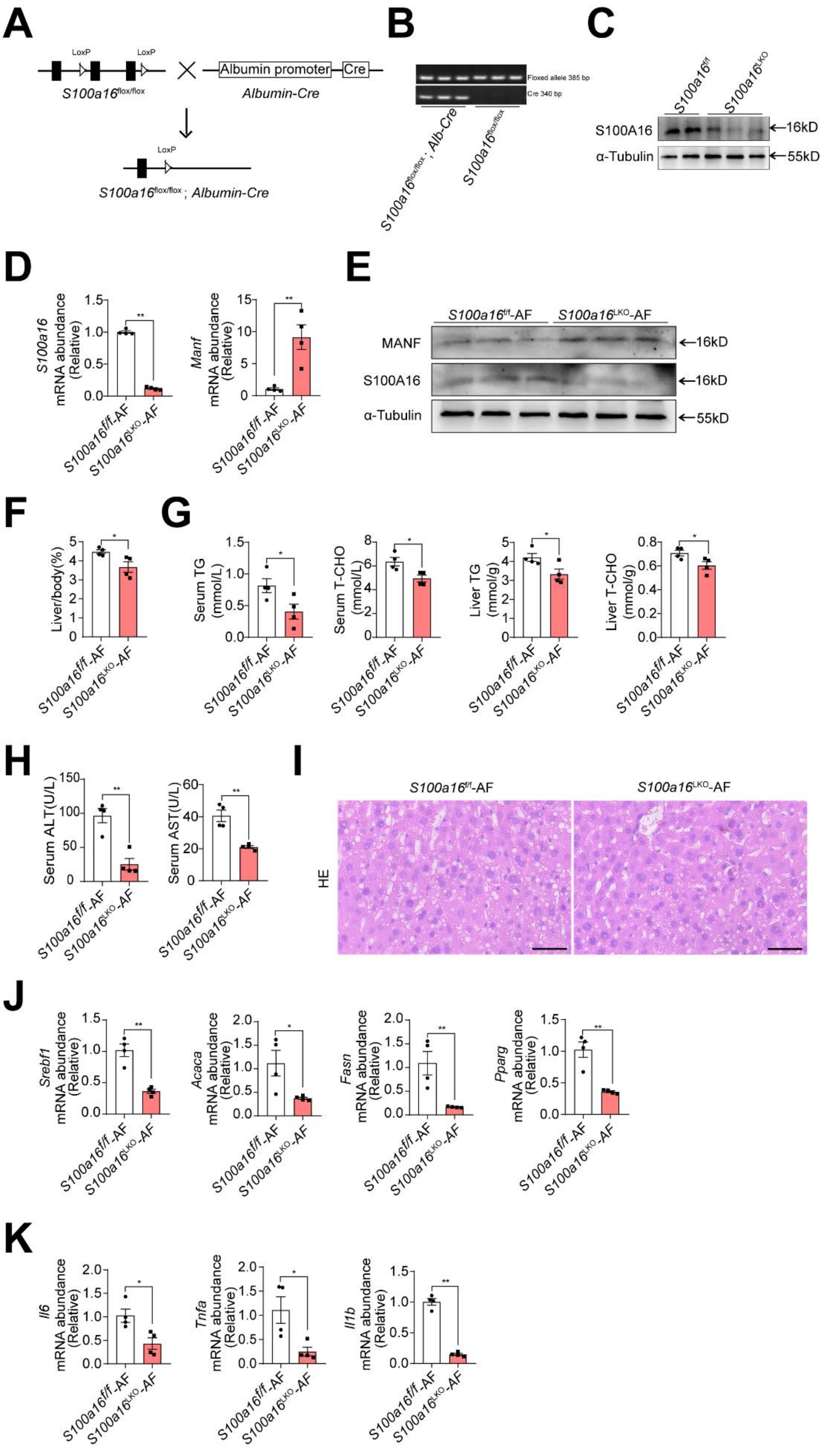
A
LoxP
LoxP
S100a16flox/flox
Albumin promoter
Cre
Albumin-Cre
LoxP
S100a16flox/flox; Albumin-Cre
B
Floxed allele 385 bp
Cre 340 bp
S100a16flox/flox; Alb-Cre
S100a16flox/flox
C
S100a16f/f
S100a16LKO
S100A16
16kD
α-Tubulin
55kD
D
S100a16 mRNA abundance (Relative)
Manf mRNA abundance (Relative)
S100a16f/f-AF
S100a16LKO-AF
E
MANF
S100A16
α-Tubulin
16kD
55kD
F
Liver/body(%)
G
Serum TG (mmol/L)
Serum T-CHO (mmol/L)
Liver TG (mmol/g)
Liver T-CHO (mmol/g)
H
Serum ALT(U/L)
Serum AST(U/L)
I
HE
J
Srebf1 mRNA abundance (Relative)
Acaca mRNA abundance (Relative)
Fasn mRNA abundance (Relative)
Pparg mRNA abundance (Relative)
K
Il6 mRNA abundance (Relative)
Tnfa mRNA abundance (Relative)
Il1b mRNA abundance (Relative)

Fig.S5 (A) The strategy for creating *S100a16* hepatocyte-specific knockout mice. (B) A representative gel electrophoresis image for genotyping. (C) Western blot analyses of S100A16 in the liver from *S100a16*$^{f/f}$ and *S100a16*^{LKO} mice. (D-K) *S100a16*$^{f/f}$ or *S100a16*^{LKO} mice were fed separately with the corresponding liquid diet 10 days and administered a single binge of ethanol. (D) mRNA and (E) protein levels of S100A16, MANF in the liver from *S100a16*$^{f/f}$ and *S100a16*^{LKO} mice. (F) Liver weight/body weight (%). (G) Serum TG, TC levels and liver TG, TC levels. (H) Serum ALT, AST levels. (I) Representative H&E staining of mice livers. Scale bars: 50 μm. (J) Relative mRNA levels of *Srebf1*, *Acaca*, *Fasn*, and *Pparg*. (K) Relative mRNA levels of *Il6*, *Tnfa*, and *Il1b*. α-Tubulin served as the loading control. All data are represented as the mean ± SEM values. *: $p < 0.05$ *vs*. the control group, **: $p < 0.01$ *vs*. the control group.

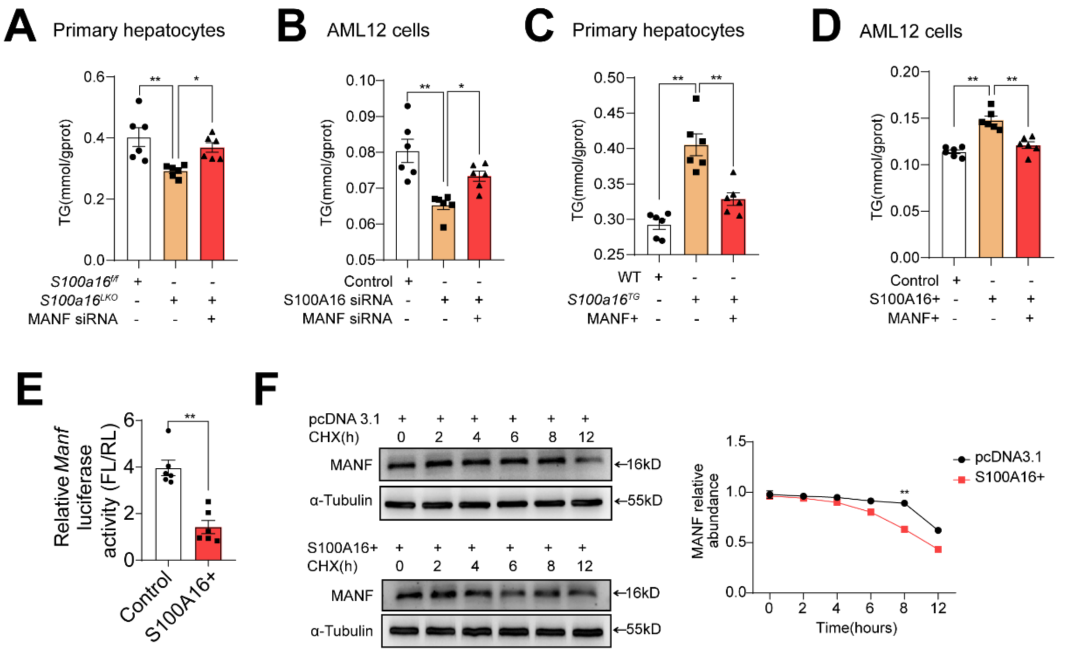

Fig.S6 (A) TG contents in ethanol-induced primary hepatocytes from *S100a16*$^{f/f}$ or *S100a16*^{LKO} mice transfected with *Manf* siRNA. (B) TG contents in ethanol-induced AML 12 cells transfected with *S100a16* siRNA and *Manf* siRNA. (C) TG contents in ethanol-induced primary hepatocytes from WT or *S100a16*^{TG} mice transfected with *Manf* overexpression plasmid. (D) TG contents in ethanol-induced AML 12 cells transfected with S100A16 and MANF overexpression plasmid. (E) Luciferase reporter gene assay for effect of S100A16 overexpression plasmid (S100A16+) on MANF gene promoter. The MANF luciferase reporter plasmid was co-transfected with S100A16 full-length or pcDNA 3.1 plasmid in AML 12 cells. The luciferase activity was normalized with renilla luciferase as the internal control. (F) MANF protein expression in AML 12 cells transfected with S100A16 overexpression or vector plasmid treated with CHX (25 μg/mL) at predict time. Quantification of western blots on the right. α-Tubulin served as the loading control. All data are represented as the mean ± SEM values. *: $p < 0.05$ *vs*. the control group, **: $p < 0.01$ *vs*. the control group.

Supplementary Tables

Table S1

The information of the primary antibodies used in this study.

Antibody	Company	Cat#	Species	Dilution
MANF	Abcam	Ab67271	rabbit	1:1000
S100A16	Proteintech	11456	rabbit	1:500
α-Tubulin	Proteintech	11224	rabbit	1:1000
GAPDH	Proteintech	HRP60004		1:5000
BIP	CST	3177	rabbit	1:1000
p-IRE1	Abcam	Ab48187	rabbit	1:1000
IER1	CST	3294	rabbit	1:1000
ATF4	Proteintech	11815	rabbit	1:500
P-elF2α	CST	3398	rabbit	1:1000
elF2α	CST	5324	rabbit	1:1000
ATF6	CST	65880	rabbit	1:1000

Table S2

The sequence information of the primers used for RT-qPCR.

Genes	Species	Forward	Reverse
S100a16	mouse	CGGACACAGGGAACCGAAAG	GTCCAGTATTCGTCAAAGCAGA
Manf	mouse	TCTGGGACGATTTTACCAGGA	TCTTGCTTCACGGCAAAACTTTA
18s	mouse	GTAACCCGTTGAACCCCATT	CCATCCAATCGGTAGTAGCG
Srebf1	mouse	GATGTGCGAACTGGACACAG	CATAGGGGGCGTCAAACAG
Fasn	mouse	GGAGGTGGTGATAGCCGGTAT	TGGGTAATCCATAGAGCCCAG
Acaca	mouse	GATGAACCATCTCCGTTGGC	GACCCAATTATGAATCGGGAGTG
Ppara	mouse	CATTTGTATGACTCATACATAAAGT	CGGATGGCCACCTCTTTGCTCTG
Il6	mouse	TAGTCCTTCCTACCCCAATTTCC	TTGGTCCTTAGCCACTCCTTC
Il1b	mouse	GCAACTGTTCCTGAACTCAACT	ATCTTTTGGGGTCCGTCAACT
Tnf	mouse	CTGAACTTCGGGGTGATCGG	GGCTTGTCACTCGAATTTTGAGA
